# Plant Essential Oils Enhance Diverse Pyrethroids against Multiple Strains of Mosquitoes and Inhibit Detoxification Enzyme Processes

**DOI:** 10.3390/insects9040132

**Published:** 2018-10-04

**Authors:** Edmund J. Norris, Jacob B. Johnson, Aaron D. Gross, Lyric C. Bartholomay, Joel R. Coats

**Affiliations:** 1 Department of Entomology, Iowa State University, Ames, IA 50011, USA; ejnorris@iastate.edu (E.J.N.); jacob14@iastate.edu (J.B.J.); 2 Department of Entomology, Virginia Polytechnic Institute and State University, Blacksburg, VA 24061, USA; adgross@vt.edu; 3 Department of Pathobiological Sciences, University of Wisconsin, Madison, WI 53706, USA; lyric.bartholomay@wisc.edu

**Keywords:** mosquito, plant essential oil, toxicology, resistance, synergism

## Abstract

Mosquito-borne diseases account for the deaths of approximately 700,000 people annually throughout the world, with many more succumbing to the debilitating side effects associated with these etiologic disease agents. This is exacerbated in many countries where the lack of mosquito control and resources to prevent and treat mosquito-borne disease coincide. As populations of mosquito species grow more resistant to currently utilized control chemistries, the need for new and effective chemical means for vector control is more important than ever. Previous work revealed that plant essential oils enhance the toxicity of permethrin against multiple mosquito species that are of particular importance to public health. In this study, we screened permethrin and deltamethrin in combination with plant essential oils against a pyrethroid-susceptible and a pyrethroid-resistant strain of both *Aedes aegypti* and *Anopheles gambiae*. A number of plant essential oils significantly enhanced the toxicity of pyrethroids equal to or better than piperonyl butoxide, a commonly used synthetic synergist, in all strains tested. Significant synergism of pyrethroids was also observed for specific combinations of plant essential oils and pyrethroids. Moreover, plant essential oils significantly inhibited both cytochrome P450 and glutathione *S*-transferase activities, suggesting that the inhibition of detoxification contributes to the enhancement or synergism of plant essential oils for pyrethroids. This study highlights the potential of using diverse plant oils as insecticide additives to augment the efficacy of insecticidal formulations.

## 1. Introduction

Synthetic insecticides have proven immeasurably valuable in the fight against economically relevant insects and pests of public-health importance. A select few have been particularly important in mosquito-borne-disease control programs in recent decades [1,2]; in particular, pyrethroids, organophosphates, carbamates, and organochlorines have been main-line defenses against mosquito-borne disease. However, resistance to these insecticides has been documented on every continent where mosquitoes are present, and will continue to challenge communities in the coming decades unless new chemistries are explored [1,3,4]. To make matters worse, insecticide-resistant mosquitoes and associated disease transmission have a disproportionately adverse impact in many developing nations [5]. 

By promoting the continued influx of sodium ions across the membrane of neurons in insects, pyrethroids cause hyperexcitability of the insect nervous system, contributing to spastic paralysis and the eventual death of the insect [6,7]. This insecticidal class has relatively low mammalian toxicity, so it can be used in close proximity to people; indeed, it is widely used in indoor residual sprays, ultralow volume applications, insecticide-treated bed nets, spatial repellents, and insecticide-treated clothing [8,9,10,11,12,13,14,15]. However, with the increase of pyrethroid-resistant mosquito populations throughout the world, this insecticidal class is becoming less effective at preventing mosquito-borne disease [12]. Because of this, the potential for mosquito-borne-disease epidemics is greater now than in previous decades [14]. 

Insecticide resistance evolves rapidly through mutations in genes that encode proteins that are targeted by insecticides or facilitate the metabolism or excretion of toxic insecticidal agents. Case in point, single-nucleotide polymorphisms in knockdown resistance (*kdr*) lead synthetic pyrethroids and DDT to have lower affinities to their molecular target, that is, voltage-gated sodium channels [6,15,16]. This, in turn, lowers their efficacy, and mosquitoes become less susceptible to higher amounts of insecticide used in the field. Upregulation of genes that encode detoxification enzymes can also confer resistance by enabling insect pest species to more effectively metabolize or remove xenobiotics from their cells and tissue [17,18,19]. Finally, alterations in the composition of the cuticle can alter insecticide uptake into the organism and then to target tissues [20]. 

To overcome insecticide resistance, insecticide synergists are commonly used to increase the bioavailability of insecticides in the insect body, allowing more insecticide to reach its molecular target. These synergists act by inhibiting various detoxification enzymes [14], thereby altering chemical metabolism or excretion. Piperonyl butoxide (PBO) is the most commonly used insecticide synergist on the market today. It acts by inhibiting monooxygenases that metabolize toxins. Although PBO is a valuable tool in synergizing the efficacy of select pyrethroids, its persistence in the environment and toxicity to mammals and other nontarget organisms are concerning. Numerous studies have demonstrated that PBO causes significant hepatotoxicity in mice and rats, is carcinogenic, and is teratogenic in mice exposed to threshold levels [21,22,23]. These effects could translate into adverse effects on humans exposed to high levels of these compounds. As such, novel insecticide development should involve formulating both new insecticidal agents and insecticide synergists. 

Plant essential oils are emerging as an attractive alternative to PBO to synergize select pyrethroids. Gross et al. characterized the combinatorial efficacy of permethrin and 35 plant essential oils against both *Aedes aegypti* and *Anopheles gambiae.* A number of plant essential oils increased the knockdown and mortality of permethrin, and this enhancement was greater than expected due to additive action [24]. It was hypothesized that plant oils were capable of enhancing permethrin toxicity via both additive and synergistic interactions, depending on the oil in question. This effect warrants further investigation, as this study highlights the potential of plant essential oils to synergize insecticides that are fundamental elements of mosquito-borne disease control programs. 

We reasoned that the utility of these formulations could be extended to enhance the efficacy of pyrethroids against pyrethroid-resistant mosquito strains, and ultimately could replace PBO in insecticide formulations. For this study, we aimed to characterize select plant essential oils to enhance the efficacy of pyrethroids against pyrethroid-resistant and pyrethroid-susceptible strains of *Ae. aegypti* and *An. gambiae.* The goal was to assess the ability of these oils to enhance the efficacy of both type I and type II pyrethroids (permethrin and deltamethrin, respectively) to represent the products currently in use. We obtained a *kdr*-resistant strain of *Ae. aegypti* (Puerto Rico) and a strain of *An. gambiae* (AKRON strain), both of which are resistant to pyrethroids [25,26,27,28,29]. Both of these strains possess multiple mechanisms that confer resistance to pyrehtroids, and may shed light on the potential of plant essential oils to enhance or synergize pyrethroids when applied against insecticide-resistant populations of mosquitoes. We compared the enhancement of pyrethroids in susceptible strains of mosquitoes to the enhancement of pyrethroids in insecticide-resistant strains to characterize the potential of plant oils to enhance this incredibly important class of control chemistry. 

To further study the ability of plant essential oils to enhance or synergize select pyrethroids, we explored the activity of select plant essential oils in vitro using model substrate detoxification enzyme activity assays. These data, coupled with the efficacy of combinatorial mixtures of both pyrethroids and plant essential oils *in vivo*, validate the use of plant essential oils as additives in insecticidal formulations to overcome insecticide resistance in two medically significant mosquito species. 

## 2. Materials and Methods

### 2.1. Mosquito Strains

Mosquitoes were obtained from BEI Resources [28]. The *Aedes aegypti* Puerto Rico strain (NR-48830) has a documented kdr phenotype based on a mutation in the voltage-gated sodium channel and enhanced detoxification capacity associated with upregulated cytochrome P450 [25,26,29]. The *Anopheles gambiae* AKRON strain (MRA-913) is resistant to carbamates, and it has been recently characterized as resistant to pyrethroids via multiple mechanisms, including upregulation of oxidative and esterase activities [27,28]. The insecticide-susceptible strains, *Anopheles gambiae* G3 and *Aedes aegypti* Liverpool, were obtained from BEI Resources and from the Parasitology/Medical Entomology Laboratory at the University of Wisconsin, respectively. 

### 2.2. Mosquito Rearing

*Ae. aegypti* and *An. gambiae* colonies were reared according to standard laboratory procedures. Briefly, adults were placed in colony cages (47 cm × 47 cm × 47 cm) and reared at constant temperature and humidity (27 °C and 70% RH). Cotton pads moistened with 10% sucrose solution were provided to mosquitoes ad libitum. Mosquitoes were provided defibrinated sheep blood (Hemostat Laboratories, Dixon, CA, USA) to promote egg laying. Eggs were collected from colony cages shortly after laying. All strains, except for *An. gambiae* G3, were supplied sheep blood using a custom glass fixture with Parafilm^®^ stretched over the bottom to serve as the presented membrane. *An. gambiae* were fed using defibrinated sheep blood; however, adult females were periodically fed using a live rabbit (*Oryctolagus cuniculus*) to maintain the vigor of the colony (The Institutional Animal Care and Use Committee (IACUC) Approval Log # was 12-2-5400-Z for the use of the rabbit for blood-feeding mosquitoes. *Ae. aegypti* pupae were separated based on size dimorphism. *An. gambiae* adult females for both strains were aspirated shortly after eclosion. This ensured that the females used for testing were virgins. Adult mosquitoes used for testing were kept in 1 pt cartons (Hihtamaki, De Soto, KS, USA) in densities of approximately 50 females per carton and provided 10% sucrose solution via moistened sucrose pads. All mosquitoes used for testing were 2–5 days old and not bloodfed. 

### 2.3. Synthetic Pyrethroids, Detoxification Enzyme Inhibitors, and Plant Essential Oils

Permethrin Z:E 40:60 (purity 98%) was obtained from EcoSMART Technologies, Inc., Roswell, GA, USA. Deltamethrin (purity 99.7%) was obtained from Sigma-Aldrich Co. LLC., St. Louis, MO, USA. Plant essential oils were provided by EcoSMART Technologies, Inc. and originally sourced by Berjé, Inc., Carteret, NJ. S,S,S-tributyl phosophorotrithioate (DEF) and diethyl maleate (DEM) were obtained from Chem Service, West Chester, PA, USA. Plant essential oils were sourced and stored in the refrigerator (4 °C) in a dark environment to prevent breakdown. This also served to prevent batch variability, which can be a significant hurdle in the efficacy testing of plant essential oils. The major components (obtained via gas chromatography-mass spectroscopy) of all of the oils used in this study can be found in Supplemental Information 1.

### 2.4. Insecticide Susceptibility Testing

Topical applications were performed to evaluate the toxicity of permethrin and deltamethrin on all four strains of mosquito in this study. For this work, we followed a modified World Health Organization (WHO) protocol [11]. In short, pyrethroids were dissolved in acetone at concentrations that would cause between 10% and 90% mortality. Mosquitoes were dosed with 0.2 μL of each solution, with 25 mosquitoes treated per replicate. For each insecticide, at least 5 concentrations were applied (as individual replicates) with a minimum of 3 biological cohorts. Treated mosquitoes were placed into a small deli cup (8 ounces) with a tulle placed over the top to prevent escape. Treated mosquitoes were provided with 10% sucrose solution via cotton pads, and maintained at a constant temperature of 27 °C, with a relative humidity of 80%, and a relative light:dark cycle of 16:8 h. A Probit model (PROC PROBIT, SAS Institute, Inc., Cary, NC, USA) was used to calculate the LD_50_ and LD_25_ values for each pyrethroid [30]. Control responses (acetone-only applications) were accounted for using the OPTC command. A minimum of 750 mosquitoes were used for each insecticide treatment. A lack of overlap among the 95% confidence intervals between two insecticides illustrated a statistically dissimilar response. 

Mosquitoes were also exposed to a discriminating dose of pyrethroid via a slightly modified World Health Organization exposure tube assay [31]. A discriminating dose of 1 mL of 0.75% permethrin solution (*w*/*v*) was applied to filter papers (12 × 15 cm). Mosquitoes for each strain were introduced into the untreated chamber and allowed to acclimate for 1 min. Mosquitoes from each strain were then gently blown into the exposure chamber (with the treated filter paper draping the lining of the walls) for 1 h, and knockdown was recorded at various time points throughout the assay (1 min, 5 min, 10 min, 15 min, 30 min, and 60 min). A nonlinear logarithmic curve was used to model the time-course knockdown response of each mosquito strain over time. 

### 2.5. Enhancement of Insecticides Applied in Concert with Plant Oils

Topical applications were performed with combinatorial mixtures of plant oil applied in conjunction with an LD_25_ of each synthetic pyrethroid. Two plant essential-oil concentrations were used for each oil, 1% *w*/*v* and 5% *w*/*v*, in combination with permethrin and deltamethrin. These concentrations were chosen to identify combinations of plant essential oils that caused less than 50% mortality. The LD_25_ of each synthetic insecticide was calculated from the PROC PROBIT model described in the previous Insecticide Susceptibility Testing section. A volume of 0.2 μL of solution of each combination was applied to 25 mosquitoes/replicate, with a minimum of 3 biological replicates used for this study. The percentage enhancement of synthetic insecticides was calculated by the following equation, as used by Gross et al. in a previous report [26]:

(1) $$\frac{\left(Percentage\;toxicity\;of\;combination\right)-\left(Percentage\;toxicity\;of\;pyrethroid\right)}{\left(Percentage\;toxicity\;of\;pyrethroid\right)}\times 100$$

Percentage enhancement values were compared to combinations of PBO, a commonly utilized insecticide synergist. PBO was applied on the two same concentrations. A two-tailed Student *t*-test was performed to assess differences between PBO and plant essential-oil enhancement at each application rate. An α of 0.05 was used to declare statistically significant differences in percentage enhancement.

### 2.6. Synergism of Pyrethroids by Plant Essential Oils

The LD_25_ of pyrethroids alone and either 1% or 5% plant essential oils alone were applied in the same experiments of applications of plant essential-oil/pyrethroid combinatorial mixtures. This allowed for the assessment of the toxicity of each individual component. These data were used to characterize the synergistic potential of plant essential oils when applied in combination with pyrethroids. To calculate synergism, we utilized the method described by Mansour et al. [32]. The cotoxicity factor of the combinatorial mixture of plant essential oils/pyrethroids was calculated using the following equation:

(2) $$\frac{\left(Observed\;\%\;mortality\right)-\left(Expected\;\%\;mortality\right)}{\left(Expected\;\%\;mortality\right)\;}\times 100$$

Observed percentage mortality was calculated for mosquitoes at 24 h post exposure, and expected mortality was calculated as the mortality caused by the pyrethroid applied alone + the mortality caused by the oil alone at each application rate (1% or 5%). Mixtures that possessed cotoxicity factors greater than 20 were defined as synergistic, and values lower than –20 were defined as antagonistic. Values between this range were defined as purely additive. 

### 2.7. Inhibition of Detoxification Enzymes

Inhibition of glutathione *S*-transferases, esterases, or cytochrome P450 enzymes by selected plant essential oils was assessed using model substrate enzyme activity assays. Methods outlined by the WHO were utilized for the assessment of esterase and glutathione s-transferase activity [33]. Acetone was the delivery vehicle for all plant essential oil/inhibitor treatments. For each treatment, mosquitoes were topically treated with 0.2 μL of 5% enzyme inhibitor (DEM or DEF) or plant essential oil in acetone 4 h prior to the homogenization of mosquitoes for assay preparation. Mosquito homogenates were maintained on ice until aliquoted into the microplate to assess enzyme activity. A minimum of 3 biological replicates were completed for each plant essential oil or inhibitor treatment (DEF or DEM). Bradford assays were used to standardize treatments, so that differences in weight among mosquito cohorts were adequately accounted. Bradford assays were performed for 3 mosquitoes for treatment in each microplate to obtain representative protein content for each replicate. Enzyme activity was reported as the normalized percentage activity (AU/RFU) compared to an acetone control. Averages of the percentage activities for each replicate were used for statistical analysis. A one-way ANOVA with a posthoc Student Newman–Keuls test (α = 0.05) were performed to assess significance between the treatment and the acetone control.

To assess α-naphthyl esterase activity, we followed the WHO guidelines with minor modifications. Mosquitoes treated with either plant essential oils or DEF (10 μg/mosquito) were homogenized in individual microcentrifuge tubes in a volume of 500 μL of 1× phosphate buffered saline (PBS). Forty microliters of this homogenate solution was introduced into each well of the microplate, followed by the addition of 40 μL of PBS and a working 1-naphthyl acetate solution. This working solution contained 3 mM naphthyl acetate with 1% SDS by weight. The final concentration of naphthyl acetate was 1.95 mM per each assay well. Microtiter plates were incubated at room temperature for 20 min, after which Fast Blue Stain was used as a counterstain and to stop the reaction. Absorbance values were recorded at 490 nM. 

To assess glutathione *S*-transferase activity, WHO guidelines were followed again but with minor changes in the protocol. Mosquitoes were treated with DEM (10 μg/mosquito) and homogenized in 500 μL of 1× PBS. A concentration of 10.6 mM reduced glutathione was made in 2.5 mL of PBS. A 63 mM solution of chlorodinitrobenzene (CDNB) in 10 mL of methanol was also made. A volume of 125 μL of CDNB was added to 2.5 mL of the reduced glutathione to produce a solution of 3 mM CDNB and 10 mM reduced glutathione. Each well contained 40 μL of homogenate, 40 μL of PBS, and 150 μL of working solution. This was incubated for 20 min and stopped with 40 uL of a 1% Fast Blue stain solution. Absorbance was recorded at 340 nM.

Monooxygenase activity was assessed by the methods previously described for the other enzyme assays, but using the protocol outlined by Anderson and Zhu (2004) with some optimization [34]. In short, mosquitoes were treated with 0.2 μL of 5% PBO or plant essential oils 24 h prior to the assay of enzyme activity. Mosquitoes were again collected and homogenized similarly to the other assay systems, and enzyme homogenate was kept on ice until each assay. Ten mosquitoes from each treatment group were collected and homogenized in 5 mL of 1× PBS. Enzyme homogenates were transferred to an ultracentrifuge tube and were centrifuged at 10,000× *g* for 20 min to remove large debris from the homogenate and isolate the microsomal fraction. The supernatant was used as the enzyme preparation. In each well, 40 uL of a working solution of 54 mM reduced NADPH and 52.5 mM 7-ethoxycoumarin were added to 80 μL of the enzyme homogenate for each treatment group. This plate was incubated in the dark for 45 min at 27 °C, after which it was removed from the incubator. In order to remove excess reduced NADPH (fluorescent in the same range as 7-hydroxycoumarin), 10 μL of 100 mM oxidized glutathione and 1.0 U glutathione reductase were added to each well, and the plate was further incubated for 1 h at 37 °C. Fluorescence was recorded at 465 nM with an excitation wavelength of 390 nM. 

## 3. Results/Discussion

### 3.1. Enhancement/Synergism

This study revealed the bioactivity of several plant essential oils to enhance various pyrethroids against both pyrethroid-susceptible and pyrethroid-resistant strains of *Ae. aegypti* and *An. gambiae.* In order to quantify the resistance of each strain to the insecticides used in this study, we compared the LD_50_ values for both permethrin and deltamethrin of the pyrethroid-susceptible strains to the pyrethroid-resistant strains (Table 1). The AKRON strain of *An. gambiae* was resistant to both permethrin and deltamethrin compared to the insecticide-susceptible G3 strain. The resistance ratio of approximately 28.4 to deltamethrin was much larger than the resistance ratio of 6 for permethrin in this strain (Akron), indicating greater fold resistance to the pyrethroid deltamethrin than permethrin. These values are similar to the values reported in the literature, with slightly different resistance ratios as compared to those reported in the previous study [27]. These differences might be attributed to different rearing conditions and differences in the subpopulations reared from the parent colony obtained from the CDC. The *Ae. aegypti* Puerto Rico strain showed significant resistance to pyrethroids; a 29-fold difference in the LD_50_ was observed compared to the susceptible Liverpool strain of *Ae. aegypti.* A lower resistance ratio of 1.8 was observed for deltamethrin. To test if resistance to pyrethroids had been lost in our colony, both Liverpool and Puerto Rico strains were challenged with natural pyrethrum extract. We observed significant resistance to pyrethrum (RR = 4.8) was still present in the Puerto Rico strain. As such, the relatively low resistance ratio to deltamethrin might be attributed to relative tolerance to this pyrethroid in the Liverpool strain. Although the Liverpool strain is not reported to be insecticide-resistant to our knowledge, nor was it continually pressured with insecticide in this study, differences might exist due to differing rearing conditions and other factors that could affect the vigor or tolerance of the mosquito strain. The LD_50_ value calculated for deltamethrin in the Puerto Rico strain is within the same order of magnitude to another reported value in the literature [13]. In fact, the LD_50_ value is higher in our report, indicating a significant resistance to deltamethrin in the Puerto Rico strain. More detailed analyses of the dose–response curves for each insecticide on each strain used in this study can be found in Supplemental Information 2. 

**Figure 1:**
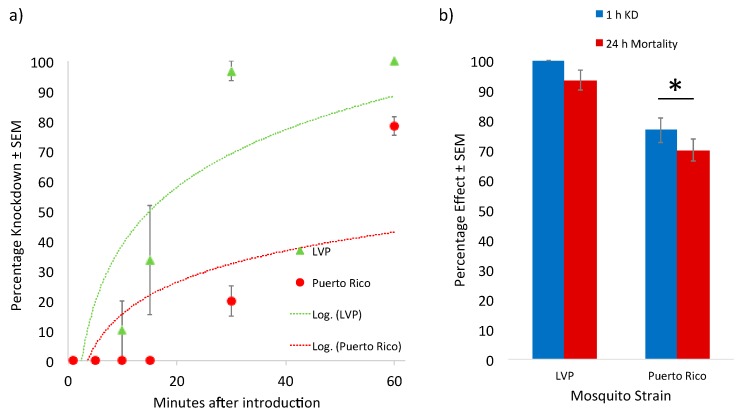
Knockdown response of *Aedes aegypti* strains exposed to a discrete dose of permethrin. (**a**) Puerto Rico strain displayed lower percentage knockdown than the Liverpool strain at all time points screened in this study. (**b**) Puerto Rico strain also displayed less percentage knockdown 1 h after removal from the exposure chamber and 24 h percentage mortality than the Liverpool strain.

**Figure 2:**
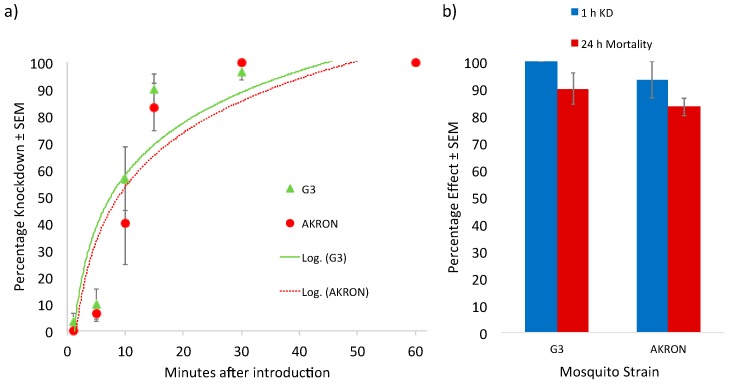
Knockdown response of *Anopheles gambiae* strains exposed to a discrete dose of permethrin. (**a**) AKRON strain experienced lower percentage knockdown than the G3 strain at all time points screened in this study. (**b**) Both strains experienced similar 1 h knockdown and 24 h mortality after removal from the exposure chamber.

The assessment of the knockdown response of these various strains challenged with permethrin also highlighted significant differences between strains. *Ae aegypti* (Liverpool) were more susceptible to permethrin knockdown as compared to *Ae. aegypti* (Puerto Rico) (Figure 1). Puerto Rico strain mosquitoes also recovered from permethrin exposure to a greater extent than Liverpool strain mosquitoes did, with a lower percentage of knockdown and mortality compared to that of the Liverpool strain at 1 h and 24 h, respectively, after being moved out of the exposure tube chamber. These differences in knockdown and 24 h mortality may be due to the presence of a *kdr*-type target site mutation and upregulated CYPs in the Puerto Rico strain compared to the Liverpool strain. The mutation in the target site may decrease the initial potency of permethrin in exposed mosquitoes. The difference in 24 h mortality may be due to increased metabolic clearance of the pyrethroid in the Puerto Rico strain compared to the Liverpool strain, leading to greater recovery after an initial insult with an insecticide. Little difference in the response to permethrin existed between the AKRON and G3 strains of *An. gambiae* (Figure 2). It is evident from this exploration that knockdown susceptibility between these strains was similar, highlighted by the overlap in the logarithmic response regression included in this dataset. No statistically significant differences in knockdown or mortality at 24 h after removal from the exposure tubes were noted between these strains. Although a resistance ratio of 6.0 was observed between these two strains in topical applications, this relatively low resistance ratio may not be large enough to detect statistically significant differences in this bioassay. Numerically lower levels of both 1 h knockdown and 24 h mortality were observed in the AKRON strain compared to the G3 strain; however, these differences were not significant.

**Figure 3:**
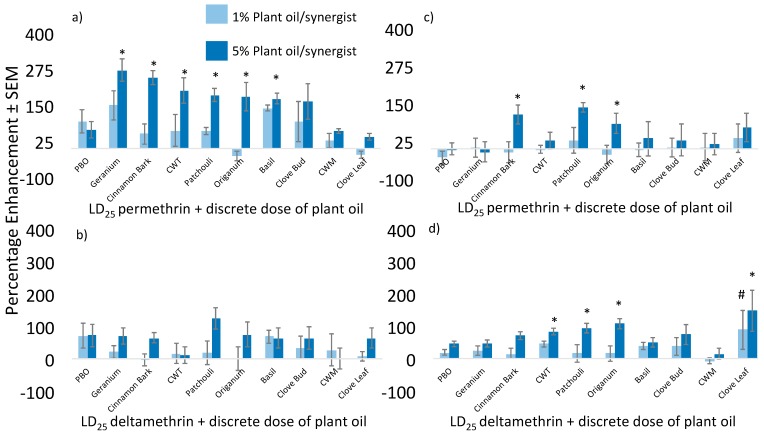
Percentage enhancement caused by plant essential oils applied in combination with select pyrethroids against two *Aedes aegypti* strains. Percentage enhancement of (**a**) permethrin + plant essential oils against the Liverpool strain; (**b**) deltamethrin + plant essential oils against the Liverpool strain; (**c**) permethrin + plant essential oils against the Puerto Rico strain; and (**d**) deltamethrin + plant essential oils against the Puerto Rico strain. Asterisks denote significantly higher percentage enhancement caused by oils applied at the 5% level than PBO applied at the same concentration. Octothorpes (#) denote significantly higher percentage enhancement caused by plant oils applied at the 1% level than PBO applied at the same concentration.

To assess the ability of plant essential oils to enhance the efficacy of various pyrethroids against these mosquito species/strains, we utilized percentage enhancement as a metric of increased insecticidal efficacy, as did Gross et al. [24]. For the *Ae. aegypti* strains screened, plant essential oils significantly increased the toxicity of both permethrin and deltamethrin (Figure 3). *p*-values associated with comparisons of percentage enhancements caused by PBO or plant oils are highlighted in Supplemental Information 3. Multiple oils outperformed PBO when applied in combination with an LD_25_ of permethrin. On the pyrethroid-susceptible Liverpool strain, geranium, cinnamon bark, cedarwood Texas (CWT), patchouli, *Origanum*, and basil showed significantly greater enhancement than a comparable application of PBO at either the 1% or 5% levels. Enhancement was less pronounced against the pyrethroid-resistant Puerto Rico strain; however, cinnamon bark, patchouli, and *Origanum* outperformed PBO at enhancing permethrin. Indeed, PBO did not enhance permethrin activity for the Puerto Rico strain. Multiple oils also positively enhanced deltamethrin against the pyrethroid-susceptible strain (Liverpool), but none enhanced deltamethrin to a greater degree than PBO (Figure 3). Patchouli oil caused higher numerical enhancement compared to PBO, but this effect was not statistically significant. Some oils did not outperform PBO, but many performed as well or better than PBO at enhancing this particular pyrethroid. This was not the case for *Ae. aegypti* (Puerto Rico), for which deltamethrin was significantly enhanced by various plant oils, and many to a greater extent than PBO. In particular, clove leaf, *Origanum*, patchouli, and CWT oils enhanced deltamethrin better than PBO when applied at either level (1% or 5%). The other oils screened performed as well as PBO at both the 1% and 5% levels when applied in combination with deltamethrin against this strain.

**Figure 4:**
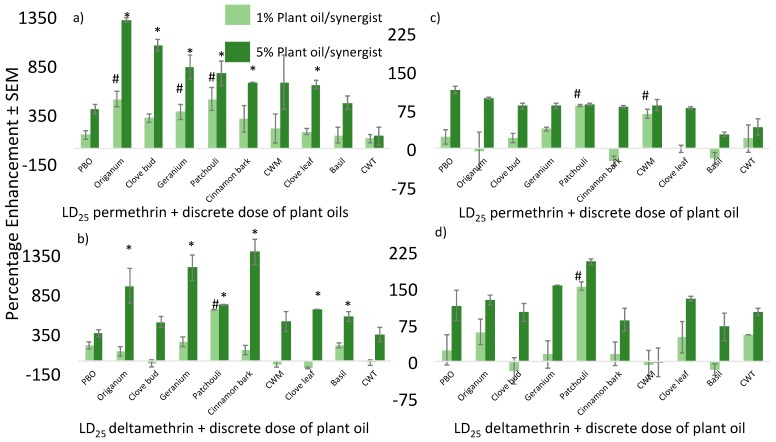
Percentage enhancement caused by plant essential oils applied in combination with select pyrethroids against two *Anopheles gambiae* strains. Percentage enhancement of (**a**) permethrin + plant essential oils against the G3 strain, (**b**) deltamethrin + plant essential oils against the G3 strain, (**c**) permethrin + plant essential oils against the AKRON strain, and (**d**) deltamethrin + plant essential oils against the AKRON strain. Asterisks denote significantly higher percentage enhancement caused by plant oils applied at the 5% level than PBO applied at the same concentration. Octothorpes (#) denote significantly higher percentage enhancement caused by plant oils applied at the 1% level than PBO applied at the same concentration.

The enhancement of pyrethroids by plant essential oils was also observed in *An. gambiae* strains. Enhancement by both PBO and plant essential oils were much higher than those for *Ae. aegypti* (LVP) and (Puerto Rico) (Figure 4). On average, the permethrin LD_25_ caused approximately 11.7% mortality at 24 h post application. This may be due to the lower percentage mortality caused by the expected LD_25_ value; lower mortality caused by the LD_25_ on the G3 strain allowed for higher detectable enhancement. This lower-than-expected mortality observed after deltamethrin with and without plant essential oils may be due to slightly more resilient or robust mosquito cohorts screened in the dose–response studies performed earlier in this project. However, this low percentage mortality should not affect the characterization of plant-oil efficacy in enhancing this pyrethroid. In the permethrin challenges, plant-oil enhancement ranged from 1310 ± 20.5% for the 5% application of *Origanum* to 100 ± 43.3% for CWT against the susceptible strain. A similar effect was noted for deltamethrin challenges with the average percentage mortality for the LD_25_ of deltamethrin across all experiments being 12.9% at 24 h. For the permethrin exposures to both strains, numerous oils outperformed PBO. 

*Origanum*, clove bud, geranium, patchouli, cinnamon bark, and clove leaf enhanced permethrin better than PBO at the 5% level in the G3 strain. Three oils, *Origanum*, geranium, and patchouli, enhanced the effect of permethrin at the 1% level against the G3 strain. For the AKRON strain, most plant oils performed as well as PBO, with a few exceptions. Basil, CWT, and cinnamon bark did not enhance permethrin as well as PBO when applied at the 5% level against this strain. Deltamethrin was significantly enhanced by a number of plant essential oils to a greater extent than PBO. *Origanum*, geranium, patchouli, cinnamon bark, and basil all caused greater enhancement than PBO at either the 1% or 5% level against the G3 strain. Again, almost all other oils performed as well as PBO with few exceptions. High enhancement values were also observed for a majority of oils applied in combination with deltamethrin against the AKRON strain. Patchouli oil significantly enhanced the efficacy of deltamethrin to a higher level than PBO against the AKRON strain at both the 1% levels. No oil outperformed PBO at enhancing permethrin or deltamethrin at the 5% level against this strain, however.

Overall, select plant oils caused percentage enhancement values that were significantly greater than PBO or equal to PBO in all combinations against both mosquitoes and all four strains. Percentage enhancement is a metric that measures the increase in toxicity of a mixture compared to the toxicant (i.e., pyrethroid) applied by itself. Percentage enhancement could be caused by inherent toxicity of the additive when applied in the mixture (i.e., PBO or plant oil). This metric was chosen to highlight the potential of plant oils to increase the insecticidal character of pyrethroids when applied in combination with select pyrethroids against multiple strains of mosquitoes. These results indicate that specific plant essential oil/pyrethroid combinations significantly enhance the efficacy of synthetic insecticides such as pyrethroids. It is likely that some of this percentage enhancement is caused by additive toxicity. A previous study highlighted that these plant essential oils are toxic to both *Ae. aegypti* and *An. gambiae* when applied via a topical-application protocol [35]. The toxicity of plant essential oils may lead to improved insecticidal mixtures, to which mosquitoes are less likely to become resistant. Multiple mechanisms of action in single insecticidal applications might lead to slowing the development of resistance [36,37].

To assess whether this enhancement was caused by synergistic interactions between the plant oils and the pyrethroids used in this study, a co-toxicity factor was calculated similar to the method outlined by Mansour et al. [32]. Plant essential oils significantly synergized pyrethroids against *Ae. aegypti* strains (Table 2 and Table 3). PBO was synergistic to the Liverpool strain of *Ae. aegypti* at the 1% level but not the 5% level. Of the plant essential oils screened, patchouli, CWT, geranium, clove bud, and cinnamon bark oils all synergized permethrin at the 1% application level. Many of these values were significantly higher than those observed for PBO. At the 5% level, PBO did not synergize permethrin against this strain, and it antagonized permethrin significantly. CWT, geranium, and cedarwood Moroccan (CWM) synergized to a much higher degree than PBO. Many oils also antagonized permethrin but produced positive enhancement percentages (Figure 3). For *Ae. aegypti* (Puerto Rico), PBO significantly antagonized the efficacy of permethrin. Patchouli produced significant synergism at the 1% application level. No other oil significantly enhanced permethrin against the Puerto Rico strain. Unexpectedly, of the oils that synergized this pyrethroid in the pyrethroid-susceptible strain, only one (patchouli oil) significantly synergized this pyrethroid in both strains. This may indicate that constituents of this oil significantly inhibits detoxification enzymes required for the clearance of permethrin in both the insecticide-susceptible and insecticide-resistant strain of *Ae. aegypti*.

Upregulated cytochrome P450 enzymes in various resistant populations of mosquitoes highlight the diverse enzyme isoforms, which may be involved in the metabolism of distinct pyrethroids from mosquitoes (4, 29, 36, 41). These different strains may also predominantly rely on distinct detoxification enzyme isoforms for clearance of the same pyrethroid. This may allow for the differential efficacy of plant oils as synergists on various strains. For deltamethrin, a similar pattern was observed. Two plant essential oils, CWM and clove leaf, successfully synergized the effect of deltamethrin on *Ae. aegypti* (Liverpool), (Table 3). Both clove leaf and CWT oils synergized deltamethrin for the pyrethroid-resistant Puerto Rico strain. This was unexpected as CWT oil did not synergize deltamethrin against the Liverpool strain. These results reveal some interesting nuances in reliance on different detoxification enzyme isoforms in the detoxification of these distinct pyrethroids. 

Numerous plant essential oils also significantly synergized permethrin against both strains of *An. gambiae* explored in this study (Table 4 and Table 5). Geranium, CWM, clove bud, and CWT significantly enhanced the efficacy of permethrin against the pyrethroid-susceptible strain of *An. gambiae*, G3. These plant essential oils synergized permethrin at the 1% level and not the 5% level. Although little synergism was observed at the higher 5% level, significant enhancement was observed for many oils (Figure 4). No plant oils synergized permethrin against the AKRON strain, but again a number of oils significantly enhanced its efficacy (Figure 4). Deltamethrin was synergized by geranium oil at the 1% and 5% level in the G3 strain. For the AKRON strain, multiple plant oils synergized deltamethrin at the 1% and 5% level. Geranium oil synergized this pyrethroid but only at the 5% level against *An. gambiae* (G3). *Origanum* and clove leaf oils also strongly synergized this pyrethroid against the AKRON strain at the 1% level. This synergism was only observed at this concentration. The lack of observed synergism at the 5% level may be due to high percentage mortality caused by these plant essential oils at this level. As was observed in *Ae. aegypti,* different plant oils synergized deltamethrin than those that synergized permethrin. If the combined percentage mortality caused by each component is too high, the ability to assess synergism is diminished. 

### 3.2. Inhibition of Detoxification Enzymes

**Figure 5:**
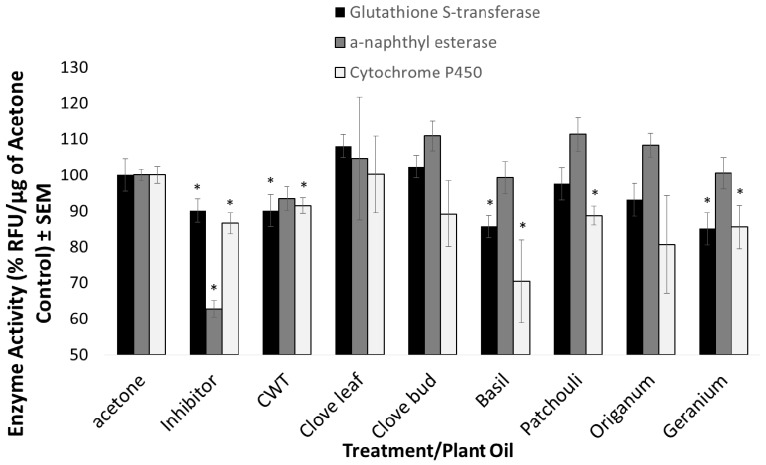
Inhibition of various detoxification enzyme systems via application of plant essential oils or synthetic inhibitors (diethyl maleate (DEM), S,S,S-tributyl phosophorotrithioate (DEF), or PBO). All synthetic inhibitors caused significant levels of inhibition of respective enzyme processes compared to the acetone control. Select plant essential oils also significantly inhibited specific enzyme systems. CWT, basil, and geranium oils significantly inhibited both glutathione s-transferase activity and cytochrome P450 activity. Glutathione *S*-transferases and cytochrome P450 enzyme systems were the most significantly inhibited by the plant essential oils screened in this study.

To explore the mechanism of synergism caused by select plant essential oils, we assessed the degree to which select plant essential oils inhibited various detoxification enzyme systems. Of the detoxification-enzyme systems screened, plant essential oils inhibited both cytochrome P450-dependent monooxygenase and glutathione *S*-transferase activity (Figure 5). All of the synthetic enzyme inhibitors utilized in this study as positive controls significantly inhibited each respective enzyme system. Of the synthetic enzyme inhibitor studies, DEF, a potent inhibitor of esterase activity, was most capable of inhibiting the α-naphthyl esterase system. Both PBO and DEM significantly inhibited cytochrome P450 activity and glutathione *S*-transferase activity compared to the control, respectively. For this study, only the oils that caused significant synergism of various pyrethroids were screened as enzyme inhibitors. Glutathione *S*-transferase activity was most significantly inhibited by geranium, basil, and CWT. Basil, geranium, patchouli, and CWT all significantly inhibited cytochrome P450 enzyme activity compared to the control. CWT and basil oil significantly inhibited glutathione *S*-transferase and cytochrome P450 activity. CWT produced significant synergism when applied in combination with permethrin against the *Ae. aegypti* Liverpool strain and the *An. gambiae* G3 strain. Therefore, CWT oil may act as a potent synergist of permethrin by inhibiting permethrin detoxification mediated by both glutathione *S*-transferase and cytochrome P450 activity. Although basil oils significantly inhibited both glutathione *S*-transferase and cytochrome P450 activity, this oil did not produce significant synergism of permethrin or deltamethrin in any of the species/strains tested in this study. The 7-ethoxycoumarin de-ethylase activity detected in this assay may be caused by distinct cytochrome P450 isoforms that are not involved in pyrethroid metabolism. It is possible that basil oil inhibits cytochrome P450 enzymes that are actively involved in 7-ethoxycoumarin de-ethylase activity but not permethrin or deltamethrin detoxification. Similarly, basil oil may inhibit glutathione *S*-transferases that are not actively involved in pyrethroid metabolism and excretion. Alternatively, only one of these enzyme processes may be more involved in the degradation of pyrethroids than the other. This exploration demonstrates the ability of plant essential oils to inhibit these processes compared to a vehicle control. Overall, plant essential oils significantly enhanced the efficacy of both type I and type II pyrethroids against susceptible and resistant strains of *Ae. aegypti* and *An. gambiae* mosquitoes. In general, more types of plant essential oils enhanced permethrin compared to deltamethrin. Moreover, the cotoxicity factors of various oils were higher in the type I pyrethroid, permethrin, compared to deltamethrin, the type II pyerthroid. Synergism of pyrethroids by plant oils was more significant and common on the two pyrethroid-susceptible strains of both *Ae. aegypti* and *An. gambiae* compared to the corresponding pyrethroid-resistant strains for each species. This is congruent with documented and multiple mechanisms for resistance involved in both strains. Estep et al. noted that numerous cytochrome P450 enzymes were significantly upregulated in *Ae. aegypti* (Puerto Rico) [25]. The increased detoxification capacity, coupled with the well-established *kdr*-type resistance of this strain may limit the ability of plant oils to significantly synergize pyrethroids against this strain. Multiple resistance mechanisms in the AKRON strain may also significantly predispose the mosquito to the toxic effect of plant essential oils. Previous studies revealed the presence of a *kdr* mutation in the AKRON strain and upregulated 7-ethoxycoumarin de-ethylase activity [27,28]. Despite lower levels of synergism observed in these resistant strains, enhancement (i.e., greater kill than the pyrethroid applied alone) was observed for most of the plant essential oils tested. In many cases, plant oils outperformed the commercially available PBO. 

Previous studies have shown that plant terpenoids enhance the toxicity of other select terpenoids. Pavela demonstrated that specific combinations of terpenoids caused significantly higher mortalities than the expected mortalities produced via additivity [38]. Moreover, other studies have demonstrated that plant essential oils significantly enhance the toxicity of synthetic insecticides against mosquito larvae [39,40]. These studies in concert suggest the potential of plant essential oil and terpenoid chemistries in enhancing insecticides for public-health vector control. Thangam and Kathiresan (1991) suggested that the inhibition of detoxification enzymes by natural products could account for these synergistic interactions [41]. Our study provides additional evidence plant oils significantly inhibit detoxification enzymes. While plant essential oils may synergize synthetic insecticides via multiple mechanisms, these results highlight that observed synergism in select mixtures of plant essential oils and synthetic insecticides is produced, at least in part, by the inhibition of detoxification enzymes.

Mosquito strains were chosen in this study to represent significant vector species and populations with varied levels and mechanistic underpinnings for insecticide resistance. The observed synergism caused by plant oils and their ability to significantly increase pyrethroid toxicity against both insecticide-resistant and insecticide-susceptible mosquito populations demonstrates the potential utility of these agents in insecticide formulations. Plant oils are a promising additive for deployment in concert with pyrethroids in the field, both because of the data shown here, and because of their inherently low level environmental or toxicological impact on nontarget organisms. For example, Olyset^®^ Plus long-lasting insecticide nets (LLIN) use PBO and were more successful against multiple species of *Anopheles* [42]. These technologies represent a viable approach for controlling vector populations, especially endophilic malaria vectors. Utilizing similar LLIN technologies with plant oils in place of PBO has potential for the successful control of resistant species of mosquito. Likewise, while PBO is not heavily used in indoor residual spray campaigns, Nikpour et al. demonstrated the potential of PBO to enhance the efficacy of deltamethrin in a field study in Iran, increasing the toxicity of deltamethrin applied to various surfaces at later time points in the study [43]. Plant oils may also be able to effectively synergize or enhance the toxicity of deltamethrin in these formulations, assuming they perform in a similar manner as topical applied mixtures. Plant essential oils are relatively volatile and may readily evaporate off a treated surface. As such, technologies aimed at incorporating them into indoor residual spray campaigns and bed nets would need to overcome this technical deployment hurdle to ensure that the plant oils in formulation would be persistent and bioavailable for long enough to successfully synergize formulations. Selecting less volatile plant oils and better formulations that diminish this volatility may be most appropriate for designing future insecticide formulations. Finally, space spraying with formulations of plant oils and pyrethroids may be relevant and efficacious in the control of various mosquito species. In these deployment scenarios, volatility would be less important and should not diminish the delivery of these agents to the target pests.

## 4. Conclusions

Numerous plant oils significantly increased the toxicity of permethrin and deltamethrin, two pyrethroids commonly utilized for the control of mosquitoes, and were capable of synergizing pyrethroids in some combinations. This synergism was concentration-dependent and primarily observed in pyrethroid-susceptible mosquito strains challenged in this study. Further optimization of insecticidal formulations that include both plant oils and pyrethroids may lead to better technologies that actively synergize or enhance pyrethroids against a wider variety of mosquito species and strains that are relevant vectors. Moreover, future work may be considered that aims to better understand the specific constituents of plant oils that are most effective in enhancing or synergizing pyrethroids against public-health vector species. This may lead to better sourcing of plant oils in future insecticide formulations or the creation of specific formulations of pyrethroids and the most bioactive single-molecule constituents of plant oils. The need for new insecticides and insecticidal formulations should be a major focus in research programs aimed at the control of neglected tropical diseases vectored by arthropods. Here, we highlighted the potential of plant essential oils to act as both enhancers and synergists, which could replace PBO in future insecticide formulations. 

## Figures and Tables

**Table 1:** LD_50_ values and resistance ratios associated with each synthetic pyrethroid screened.

Compound	*n*	Species	Strain	LD_50_, μg/g (95% CI)	RR
Permethrin	1350	*Anopheles gambiae*	G3	0.632 (0.15–1.5)	
	1100	*Anopheles gambiae*	AKRON	3.8 (1.9–5.8)	6.0
Deltamethrin	875	*Anopheles gambiae*	G3	0.0026 (0.001–0.004)	
	1425	*Anopheles gambiae*	AKRON	0.074 (0.06–0.09)	28.4
Permethrin	1300	*Aedes aegypti*	Liverpool	0.42 (0.31–0.51)	
	1100	*Aedes aegypti*	Puerto Rico	12.3 (9.6–15.6)	29.3
Deltamethrin	1525	*Aedes aegypti*	Liverpool	0.34 (0.16–1.5)	
	1100	*Aedes aegypti*	Puerto Rico	0.601 (0.4–1.03)	1.8
Natural pyrethrins	850	*Aedes aegypti*	Liverpool	1.64 (0.8–2.4)	
	700	*Aedes aegypti*	Puerto Rico	7.91 (6.1–11)	4.8

**Table 2:** Synergism caused by plant essential oils in combination with permethrin against *Ae. aegypti* strains.

Strain	Synergist/Plant Oil	1% Plant Oil/Synergist						5% Plant Oil/Synergist				
		Permethrin	Synergist/Oil Alone	Expected Mortality	Observed Mortality	Cotoxicity Factor		Permethrin	Synergist/Oil Alone	Expected Mortality	Observed Mortality	Cotoxicity Factor
Liverpool	PBO	21.1	2	23.1	36	55.84		21.1	22.3	43.4	32.7	−24.65
	Patchouli	26.7	6.7	33.4	42.7	27.84		26.7	60	86.7	76	−12.34
	CWT ^a^	21	1	22	34	54.55		21	1	22	63	186.36
	Geranium	12.8	0	12.8	29.6	131.25		12.8	17.3	30.1	43.2	43.52
	CWM ^b^	34	2	36	43	19.44		34	2	36	54	50.00
	Clove Bud	24	4	28	36	28.57		24	35	59	48	−18.64
	Cinnamon Bark	18.4	4	22.4	27.2	21.43		18.4	36	54.4	61.6	13.24
	Basil	33.3	60	93.3	80	−14.26		33.3	92	125.3	90.6	−27.69
	*Origanum*	28.9	22	50.9	28.9	−43.22		28.9	54	82.9	74.4	−10.25
	Clove Leaf	48.6	2	50.6	38.9	−23.12		48.6	50	98.6	66.3	−32.76
												
Puerto Rico	PBO	34	3	37	25	−32.43		34	28	62	33	−46.77
	Patchouli	35	8	43	60	39.53		35	48	83	83	0.00
	CWT ^a^	30	1	31	29	−6.45		30	4	34	38	11.76
	Geranium	53	1	54	55	1.85		53	15	68	48	−29.41
	CWM ^b^	27	13	40	28	−30.00		27	55	82	31	−62.20
	Clove Bud	45	4	49	47	−4.08		45	79	124	57	−54.03
	Cinnamon Bark	31	4	35	28	−20.00		31	71	102	64	−37.25
	Basil	37	41	78	36	−53.85		37	11	48	49	2.08
	*Origanum*	35	2	37	29	−21.62		35	38	73	63	−13.70
	Clove Leaf	34	2	36	46	27.78		34	49	83	58	−30.12

^a^ Cedarwood Texas Oil. ^b^ Cedarwood Moroccan Oil.

**Table 3:** Synergism caused by plant essential oils in combination with deltamethrin against *Ae. aegypti* strains.

Strain	Synergist/Plant Oil	1% Plant Oil/Synergist						5% Plant Oil/Synergist				
		Deltamethrin	Synergist/Oil Alone	Expected Mortality	Observed Mortality	Cotoxicity Factor		Deltamethrin	Synergist/Oil Alone	Expected Mortality	Observed Mortality	Cotoxicity Factor
Liverpool	PBO	26	3	29	43	48.28		26	14	40	43	7.50
	CWM ^b^	29	1	30	37	23.33		30	14	44	30	−31.82
	Clove Leaf	22	2	24	29	20.83		23	42	65	43	−33.85
	Geranium	28	2	30	34	13.33		28	18	46	46	0.00
	Clove Bud	29	2	31	33	6.45		29	30	59	39	−33.90
	*Origanum*	31	0	31	31	0.00		31	38	69	52	−24.64
	CWT ^a^	27	5	32	31	−3.13		27	5	32	30	-6.25
	Patchouli	31	7	38	36	−5.26		31	43	74	65	−12.16
	Cinnamon Bark	27	3	30	26	−13.33		27	36	63	43	−31.75
	Basil	35	48	83	44	−46.99		35	63	98	43	−56.12
												
Puerto Rico	PBO	43	2	45	55	22.22		43	24	67	67	0.00
	CWM ^b^	54	4	58	50	−13.79		54	54	108	62	−42.59
	Clove Leaf	20	2	22	37	68.18		20	47	67	53	−20.90
	Geranium	41	4	45	51	13.33		41	13	54	60	11.11
	Clove Bud	35	4	39	47	20.51		35	74	109	65	−40.37
	*Origanum*	33	22	55	37	−32.73		33	49	82	66	−19.51
	CWT ^a^	38	1	39	55	41.03		38	3	41	70	70.73
	Patchouli	33	8	41	41	0.00		33	46	79	63	−20.25
	Cinnamon Bark	38	8	46	49	6.52		38	64	102	75	−26.47
	Basil	45	13	58	62	6.90		45	39	84	67	−20.24

^a^ Cedarwood Texas Oil. ^b^ Cedarwood Moroccan Oil.

**Table 4:** Synergism caused by plant essential oils in combination with permethrin against *An. gambiae* strains.

Strain	Synergist/Plant Oil	1% Plant Oil/Synergist						5% Plant Oil/Synergist				
		Permethrin	Synergist/Oil Alone	Expected Mortality	Observed Mortality	Cotoxicity Factor		Permethrin	Synergist/Oil Alone	Expected Mortality	Observed Mortality	Cotoxicity Factor
G3	PBO	13.3	24.6	37.9	30.7	−19.00		13.3	72	85.3	64	−24.97
	Geranium	8.8	13.6	22.4	41.6	85.71		8.8	72	80.8	81.6	0.99
	CWM ^b^	6.6	2.7	9.3	12	29.03		6.7	48	54.7	42.6	−22.12
	Clove Bud	8.8	16	24.8	31.2	25.81		8.8	77.6	86	85.6	−0.93
	CWT ^a^	9.3	6.7	16	20	25.00		9.3	64.7	74	75.3	1.76
	Patchouli	17.3	78.6	95.9	90.7	−5.42		17.3	97.3	114.6	98.7	−13.87
	Clove Leaf	16	54.7	70.7	60	−15.13		16	86.7	102.7	84	−18.21
	*Origanum*	12	44.7	56.7	44	−22.40		12	95.3	107.3	98	−8.67
	Cinnamon Bark	13.6	29.6	43.2	30.4	−29.63		13.6	81.6	95.2	100	5.04
	Basil	12.8	36.8	49.6	32	−35.48		12.8	95	107.8	85.6	−20.59
												
Akron	PBO	44.4	24	68.4	53.7	−21.49		44.4	47	91.4	92	0.66
	Geranium	56	13.3	69.3	76	9.67		56	30.7	86.7	100	15.34
	CWM ^b^	59	28	87	92	5.75		59	48	107	100	−6.54
	Clove Bud	56	2.7	58.7	66.7	13.63		56	77.3	133.3	100	−24.98
	CWT ^a^	59	22.7	81.7	68	−16.77		59	85.3	144.3	79	−45.25
	Patchouli	59	68	127	99	−22.05		59	96	155	100	−35.48
	Clove Leaf	56	5.3	61.3	54.7	−10.77		56	82.7	138.7	97.3	−29.85
	*Origanum*	52	4	56	49.3	−11.96		52	76	128	98.7	−22.89
	Cinnamon Bark	56	37.3	93.3	42.7	−54.23		56	96	152	98.7	−35.07
	Basil	56	21.3	77.3	45.3	−41.40		56	50.7	106.7	70.7	−33.74

^a^ Cedarwood Texas Oil. ^b^ Cedarwood Moroccan Oil.

**Table 5:** Synergism caused by plant essential oils in combination with deltamethrin against *An. gambiae* strains.

Strain	Synergist/Plant Oil	1% Plant Oil/Synergist						5% Plant Oil/Synergist				
		Deltamethrin	Synergist/Oil Alone	Expected Mortality	Observed Mortality	Cotoxicity Factor		Deltamethrin	Synergist/Oil Alone	Expected Mortality	Observed Mortality	Cotoxicity Factor
G3	PBO	12.5	18	30.5	31.5	3.28		12.5	49	61.5	45.5	−26.02
	Geranium	6.7	10.6	17.3	22.7	31.21		6.7	62.7	69.4	85.3	22.91
	Patchouli	18.4	92	110.4	91.2	−17.39		18.4	97	115.4	97.6	−15.42
	Basil	13.3	8	21.3	20	−6.10		13.3	45.3	58.6	44	−24.91
	Cinnamon Bark	6.7	26.7	33.4	16	−52.10		6.7	77.3	84	98.7	17.50
	*Origanum*	10.7	21.3	32	23.2	−27.50		10.7	80	90.7	96	5.84
	Clove Leaf	14.7	6.7	21.4	53	147.66		6.7	85.3	92	84	−8.70
	Clove Bud	17.6	17.6	35.2	13.6	−61.36		17.6	81.6	99.2	80	−19.35
	CWT ^a^	16	13.6	29.6	14	−52.70		16	72.8	88.8	49.3	−44.48
	CWM ^b^	9.3	8	17.3	6	−65.32		9.3	63.3	72.6	49.3	−32.09
												
Akron	PBO	45.5	27	72.5	62	−14.48		45.5	49	94.5	94	−0.53
	Geranium	43	11	54	48	−11.11		43	39	82	100	21.95
	Patchouli	36	74	110	79	−28.18		36	99	135	93	−31.11
	Basil	42.4	29	71.4	37.6	−47.34		42.4	49	91.4	63.2	−30.85
	Cinnamon Bark	49.1	33	82.1	55.4	−-32.52		49.1	96	145.1	84.5	−41.76
	*Origanum*	43	2.6	45.6	65	42.54		43	84	127	89	−29.92
	Clove Leaf	45.7	4	49.7	62.8	26.36		45.7	81.3	127	90.9	−28.43
	Clove Bud	47.2	17.3	64.5	39.2	−39.22		47.2	76	123.2	88	−28.57
	CWT ^a^	52	22.7	74.7	69.3	−7.23		52	89.3	141.3	84	−40.55
	CWM ^b^	47.3	27	74.3	44.7	−39.84		47.3	54	101.3	46.7	−53.90

^a^ Cedarwood Texas Oil. ^b^ Cedarwood Moroccan Oil.

## Supplementary Materials

The following are available online at http://www.mdpi.com/2075-4450/9/4/132/s1.
