# Supplementary material for: Plant Essential Oils Enhance Diverse Pyrethroids against Multiple Strains of Mosquitoes and Inhibit Detoxification Enzyme Processes
Source: Insects. 2018 Oct 4;9(4):132. doi: 10.3390/insects9040132 (PMC6316883; doi:10.3390/insects9040132)
Supplement: Supplementary file 1 [file insects-09-00132-s001.zip › Supplemental Information 2.docx]

Supplemental Information 2. Outputs for probit analyses for each insecticidal challenge.

| **Insecticide** | **Strain** | **Species** | **N** | **LD_25_ (μg/g mosquito)** | **LD_50_ (μg/g mosquito)** | **Slope (SE)** | **χ^2^ (DF)** | **95% Confidence Interval** |
| --- | --- | --- | --- | --- | --- | --- | --- | --- |
| Permethrin | Liverpool | *Aedes aegypti* | 1300 | 0.19 | 0.42 | 1.95 (0.29) | 130.2 (43) | 0.31-0.51 |
|  | Puerto Rico | *Aedes aegypti* | 1100 | 5.35 | 12.3 | 1.86 (0.31) | 136.6 (36) | 9.6-15.6 |
| Deltamethrin | Liverpool | *Aedes aegypti* | 1525 | 0.01 | 0.34 | 0.45 (0.08) | 122.2 (50) | .16-1.5 |
|  | Puerto Rico | *Aedes aegypti* | 1100 | 0.125 | 0.601 | 0.99 (0.18) | 131.1 (36) | 0.4-1.03 |
| Natural Pyrethrins | Liverpool | *Aedes aegypti* | 850 | 0.67 | 1.64 | 1.74 (0.26) | 56.9 (27) | 0.8-2.4 |
|  | Puerto Rico | *Aedes aegypti* | 700 | 3.8 | 7.91 | 2.12 (0.46) | 74 (20) | 6.1-11 |
| Permethrin | G3 | *Anopheles gambiae* | 1350 | 0.23 | 0.632 | 1.6 (0.16) | 122.2 (50) | 0.15-1.5 |
|  | AKRON | *Anopheles gambiae* | 1100 | 1.23 | 3.8 | 1.3 (0.21) | 139.02 (38) | 1.9-5.8 |
| Deltamethrin | G3 | *Anopheles gambiae* | 875 | 0.00071 | 0.0026 | 1.18 (0.22) | 93.2 (29) | 0.001-0.004 |
|  | AKRON | *Anopheles gambiae* | 1425 | 0.03 | 0.074 | 1.98 (0.22) | 125 (50) | 0.06-0.09 |
