# Supplementary material for: Plant Essential Oils Enhance Diverse Pyrethroids against Multiple Strains of Mosquitoes and Inhibit Detoxification Enzyme Processes
Source: Insects. 2018 Oct 4;9(4):132. doi: 10.3390/insects9040132 (PMC6316883; doi:10.3390/insects9040132)
Supplement: Supplementary file 1 [file insects-09-00132-s001.zip › Supplemental Information 3.docx]

| ***Aedes aegypti* (Liverpool) - Permethrin Treatment** | | |
| --- | --- | --- |
|  | **1% comparisons** | **5% comparisons** |
| **Oil** | **P-Value (PBO vs. Oil Enhancement)** | **P-Value (PBO vs. Oil Enhancement)** |
| Geranium | 0.39192475 | **0.000375258** |
| Cinnamon Bark | 0.47640737 | **0.000509806** |
| CWT | 0.653388222 | **0.01493685** |
| Patchouli | 0.649315696 | **0.031615587** |
| Origanum | 0.076808281 | **0.036094383** |
| Basil | 0.528539473 | **0.048990777** |
| Clove Bud | 0.98848789 | 0.108942504 |
| CWM | 0.312320408 | 0.91611587 |
| Clove Leaf | 0.02650477 | 0.448685692 |
|  |  |  |
| ***Aedes aegypti* (Puerto Rico) - Permethrin Treatment** | | |
|  | **1% comparisons** | **5% comparisons** |
| **Oil** | **P-Value (PBO vs. Oil Enhancement)** | **P-Value (PBO vs. Oil Enhancement)** |
| Geranium | 0.442262623 | 0.815941538 |
| Cinnamon Bark | 0.669864313 | **0.010949013** |
| CWT | 0.362952417 | 0.410848412 |
| Patchouli | 0.229940296 | **0.000985424** |
| Origanum | 0.801948207 | **0.044780172** |
| Basil | 0.507828527 | 0.479637878 |
| Clove Bud | 0.397301539 | 0.540183541 |
| CWM | 0.472859038 | 0.657250934 |
| Clove Leaf | 0.209668216 | 0.145112921 |
|  |  |  |
| **Aedes aegypti (Liverpool) - Deltamethrin Treatment** | | |
|  | **1% comparisons** | **5% comparisons** |
| **Oil** | **P-Value (PBO vs. Oil Enhancement)** | **P-Value (PBO vs. Oil Enhancement)** |
| Geranium | 0.35754719 | 0.958030772 |
| Cinnamon Bark | 0.218962112 | 0.876228906 |
| CWT | 0.302015131 | 0.197162411 |
| Patchouli | 0.466889779 | 0.429611025 |
| Origanum | 0.268490596 | 0.964101356 |
| Basil | 0.969888315 | 0.884412548 |
| Clove Bud | 0.512442516 | 0.836482717 |
| CWM | 0.494264101 | 0.186231466 |
| Clove Leaf | 0.294372843 | 0.885454002 |
|  |  |  |
| **Aedes aegypti (Puerto Rico) - Deltamethrin Treatment** | | |
|  | **1% comparisons** | **5% comparisons** |
| **Oil** | **P-Value (PBO vs. Oil Enhancement)** | **P-Value (PBO vs. Oil Enhancement)** |
| Geranium | 0.731521479 | 0.959291014 |
| Cinnamon Bark | 0.895146906 | 0.107695558 |
| CWT | 0.067712202 | **0.014978933** |
| Patchouli | 0.919759746 | **0.013194088** |
| Origanum | 0.873910561 | **0.00280492** |
| Basil | 0.210674074 | 0.856597018 |
| Clove Bud | 0.403748401 | 0.253077113 |
| CWM | 0.067483109 | 0.099379639 |
| Clove Leaf | **0.037947064** | **0.007532554** |
|  |  |  |
|  |  |  |
|  |  |  |
|  |  |  |
| ***Anopheles gambiae* (G3) - Permethrin Treatment** | | |
|  | **1% comparisons** | **5% comparisons** |
| **Oil** | **P-Value (PBO vs. Oil Enhancement)** | **P-Value (PBO vs. Oil Enhancement)** |
| origanum | **0.014901035** | **8.5096E-09** |
| clove bud | 0.078357579 | **9.59049E-06** |
| geranium | **0.022685487** | **0.00650973** |
| patchouli | **0.000136595** | **0.001400269** |
| cinnamon bark | 0.950875175 | **0.000597736** |
| CWM | 0.628827313 | 0.202237824 |
| clove leaf | 0.562020177 | **0.004018555** |
| Basil | 0.187541771 | 0.471887255 |
| CWT | 0.688911487 | **1.11641E-05** |
|  |  |  |
| ***Anopheles gambiae* (AKRON) - Permethrin Treatment** | | |
|  | **1% comparisons** | **5% comparisons** |
| **Oil** | **P-Value (PBO vs. Oil Enhancement)** | **P-Value (PBO vs. Oil Enhancement)** |
| origanum | 0.359391845 | 0.189064113 |
| clove bud | 0.92174319 | **0.021745516** |
| geranium | 0.503523905 | **0.021745516** |
| patchouli | **0.007826316** | **0.015498517** |
| cinnamon bark | 0.060613449 | **0.015402692** |
| CWM | **0.041846349** | **0.011266728** |
| clove leaf | 0.28941134 | **0.010608414** |
| Basil | 0.090734671 | **2.41484E-05** |
| CWT | 0.895350416 | **0.000342972** |
|  |  |  |
| **Anopheles gambiae (G3) - Deltamethrin Treatment** | | |
|  | **1% comparisons** | **5% comparisons** |
| **Oil** | **P-Value (PBO vs. Oil Enhancement)** | **P-Value (PBO vs. Oil Enhancement)** |
| origanum | 0.28499664 | **0.009260591** |
| clove bud | **0.004650503** | 0.087727471 |
| geranium | 0.622866993 | **4.6245E-05** |
| patchouli | **6.05481E-06** | **3.29129E-05** |
| cinnamon bark | 0.472923592 | **1.40636E-07** |
| CWM | **0.002117056** | 0.229841204 |
| clove leaf | **0.003480328** | **0.001879521** |
| Basil | 1 | **0.022355065** |
| CWT | **0.003015992** | 0.879452963 |
|  |  |  |
| **Anopheles gambiae (AKRON) - Deltamethrin Treatment** | | |
|  | **1% comparisons** | **5% comparisons** |
| **Oil** | **P-Value (PBO vs. Oil Enhancement)** | **P-Value (PBO vs. Oil Enhancement)** |
| origanum | 0.479040931 | 0.814628788 |
| clove bud | 0.3710524 | 0.740963166 |
| geranium | 0.852013538 | 0.3877831 |
| patchouli | **0.018656902** | 0.08093076 |
| cinnamon bark | 0.842297027 | 0.473764089 |
| CWM | 0.51147144 | **0.022744619** |
| clove leaf | 0.589701861 | 0.711545476 |
| Basil | 0.366345025 | 0.358402867 |
| CWT | 0.589059972 | 0.791576234 |
